# GENDER DIFFERENCES IN DISEASE BURDEN AND QUALITY-OF-LIFE TRAJECTORIES AMONG OLDER ADULTS LIVING WITH MULTIMORBIDITY

**DOI:** 10.1093/geroni/igae098.3150

**Published:** 2024-12-31

**Authors:** Arum Lim, Chitchanok Benjasirisan, Jordan Tebay, Cheryl Dennison Himmelfarb, Patricia Davidson, Binu Koirala

**Affiliations:** Johns Hopkins University, Baltimore, Maryland, United States; Johns Hopkins University, Baltimore, Maryland, United States; Johns Hopkins University, Baltimore, Maryland, United States; Johns Hopkins University, Baltimore, Maryland, United States; University of Wollongong, Wollongong, New South Wales, Australia; Johns Hopkins University, Baltimore, Maryland, United States

## Abstract

Heart failure (HF) is a leading cause of hospitalization and is associated with higher multimorbidity. Despite gender disparities and differences in HF research participation and health outcomes, differences in the trajectories of their lives have not been studied enough. This study aimed to examine the 1-year trajectories of disease burden and quality of life (QoL) for older adults with multimorbidity. Multimorbidity was defined as the presence of HF and one or more conditions among common chronic conditions in older adults. The study prospectively collected socio-demographic, clinical, and outcome data at baseline, 30-day, and 1-year follow-up using an electronic patient portal among adults aged 50 years and older living with multimorbidity. Disease burden and QoL data were collected using a modified disease burden impact scale and the EuroQoL (EQ)-5D-5L. Linear mixed-effect models were used for statistical analyses. A total of 200 patients (102 women and 98 men) completed surveys at the three time points. Participants’ mean±SD age was 71±9.5 years; men were older than women. The disease burden decreased at the 30-day follow-up (23.7±14.9) and the 1-year follow-up (24.6±15.7) compared to baseline (26.3±16.3). In an unadjusted analysis, disease burden was higher in women than men (beta=2.516; p=0.017). QoL was not significantly different by gender and time. After adjusting for age and social support, there were no significant gender differences in disease burden and QoL trajectories and no time and gender interaction. There is a need for gender-based research on multimorbidity to promote health equity.

